# Multiclass datasets expand neural network utility: an example on ankle radiographs

**DOI:** 10.1007/s11548-023-02839-9

**Published:** 2023-02-02

**Authors:** Suam Kim, Philipp Rebmann, Phuong Hien Tran, Elias Kellner, Marco Reisert, David Steybe, Jörg Bayer, Fabian Bamberg, Elmar Kotter, Maximilian Russe

**Affiliations:** 1 Department of Diagnostic and Interventional Radiology, Faculty of Medicine, Medical Center–University of Freiburg, Hugstetter Str. 55, 79106 Freiburg, Germany; 2 Department of Medical Physics, Faculty of Medicine, Medical Center–University of Freiburg, University of Freiburg, Freiburg, Germany; 3 Department of Oral and Maxillofacial Surgery, Faculty of Medicine, Medical Center–University of Freiburg, Freiburg, Germany; 4 Department of Trauma and Orthopaedic Surgery, Schwarzwald-Baar Hospital, Villingen-Schwenningen, Germany

**Keywords:** Neural network, Deep learning, Radiographs, Musculoskeletal

## Abstract

**Purpose:**

Artificial intelligence in computer vision has been increasingly adapted in clinical application since the implementation of neural networks, potentially providing incremental information beyond the mere detection of pathology. As its algorithmic approach propagates input variation, neural networks could be used to identify and evaluate relevant image features. In this study, we introduce a basic dataset structure and demonstrate a pertaining use case.

**Methods:**

A multidimensional classification of ankle x-rays (*n* = 1493) rating a variety of features including fracture certainty was used to confirm its usability for separating input variations. We trained a customized neural network on the task of fracture detection using a state-of-the-art preprocessing and training protocol. By grouping the radiographs into subsets according to their image features, the influence of selected features on model performance was evaluated via selective training.

**Results:**

The models trained on our dataset outperformed most comparable models of current literature with an ROC AUC of 0.943. Excluding ankle x-rays with signs of surgery improved fracture classification performance (AUC 0.955), while limiting the training set to only healthy ankles with and without fracture had no consistent effect.

**Conclusion:**

Using multiclass datasets and comparing model performance, we were able to demonstrate signs of surgery as a confounding factor, which, following elimination, improved our model. Also eliminating pathologies other than fracture in contrast had no effect on model performance, suggesting a beneficial influence of feature variability for robust model training. Thus, multiclass datasets allow for evaluation of distinct image features, deepening our understanding of pathology imaging.

**Supplementary Information:**

The online version contains supplementary material available at 10.1007/s11548-023-02839-9.

## Introduction

Artificial intelligence (AI) via neural networks is becoming an increasingly important tool in diagnostic imaging. Its accuracy in detecting pathologies has been increased to and even beyond the level of human radiologists [1–5]. Because of its consistency, reproducibility, speed as well as resistance to bias and distractors, a well-trained neural network has the potential to assist not only in diagnostics [6, 7], but in research as well, by reducing human effort in analyzing increasingly large amounts of data. With its ability to be attuned to well defined direct image features, indirect derivatives thereof or a combination of both, its usage could provide insight into the process of radiological pathology detection and recognition underlying the network training.

Application of neural networks in computer vision is widely established on two-dimensional real world images [8], allowing for direct adaptation onto two-dimensional radiographs. Even given their limited sensitivity, due to low radiation dose, wide availability and the simple imaging process, conventional radiographs remain the main screening and diagnostic tool in isolated musculoskeletal trauma. Routine evaluation by neural networks therefore is the logical step of technological advancement for this modality.

In this study, we present a multiclass image dataset of ankle x-ray examinations as well as improvements in image preprocessing and augmentation on the performance of a modified Inceptionv3-network in a clinical image classification task. The dataset consists of ankle x-ray images taken over 6 years at a single medical center, its intended primary purpose is to be used as a training set for a variety of deep learning algorithms in clinical application with a focus on trauma diagnostics. Its multiclass design enables an analysis of influencing factors as well as performance optimization, as we demonstrate on a fracture prediction task.

## Methods

### Study population and dataset

**Fig. 1:**
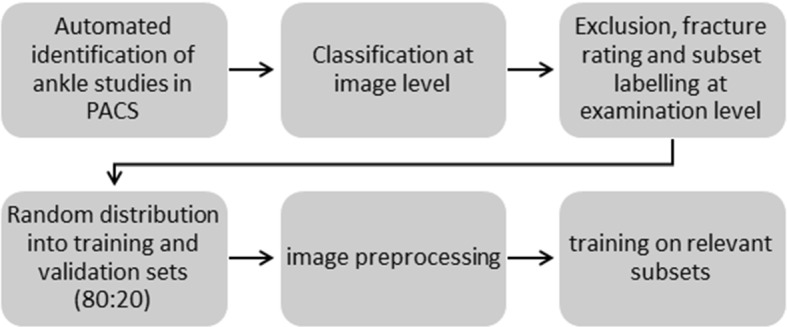
Schematic workflow for dataset creation and training

The dataset included x-ray examinations of the ankle region from adult patients at the trauma center of our institution between 2017 and 2019. In order to balance the training datasets regarding the proportion of fracture-containing examinations (from here on referred to as fracture-positive in contrast to fracture-negative ones, which were devoid of fractures), fracture-positive ankle examinations from the years 2013 to 2016 were selectively included. The image data were imported from PACS into the NORA application for further processing. NORA is a software designed to improve the connection between research and clinics in the field of medical imaging and offers customized integrated solutions to visualize and process medical imaging data [9]. The study was approved by the local ethics committee (reference: 570/19), written informed consent was waived. We minimized initial examination preselection by manually excluding only unique anatomical or post-therapeutic variations as well as gross deviations from standard image projections. In total, we imported 1,493 examinations for further network training and testing (Fig. 1).

Images were then classified into anteroposterior (ap) and lateral projections of the ankle, disregarding mortise projections for their lack of prevalence in routine ankle trauma workup at our institution. Exclusion due to clinical reasons was not performed, neither did we exclude examinations with osteosynthetic materials, prostheses, obstructed images or those displaying pathologies other than ankle fractures, but rather classified them into respective selective and partially overlapping training subsets.

### Rating and annotation

Aside from image classification according to projection, data was also classified at examination level using an NORA-embedded custom rating tool to rate relevant aspects. These included image quality, existence and graded certainty of fracture, plaster casts, internal foreign bodies, joint configuration, previous surgery, other pathologies of the bones as well as soft tissue lesions and swelling (see suppl. Table 1 and Table 1).

**Table 1:** Ankle fracture dataset

Fracture class	Image feature	Examinations
0	No fracture	1157
1	Certain fracture (certain radiographic feature like discontinuity or fragment in either projection)	236
2	Faint fracture (faint fracture line or cortical irregularity)	65
3	Doubtful or avulsed fracture (uncertain fragment, possibly ossicle)	35

Nominal fracture classes, discernibility and number of examinations for each class. Fracture class 3 was included because of frequent uncertainty regarding ossicles at subfibular and subtibial positions

Ratings were performed in consensus reading by two raters [SK, specialist trainee radiologist, MR, musculoskeletal sub-specialized board-certified radiologist] under consideration of the final examination reports, which had been created and reviewed at the time of examination by a specialist trainee and board-certified specialist of our institution, respectively. Reading error inherent to projection imaging was addressed in case of multiple examinations per patient by considering patient history as well as rating the images for repeat and follow-up examination for each patient in succession to allow for the possibility of later demarcation of fracture lines to accurately assess doubtful fracture aspects. While using all available supportive information in this way for optimized interpretation of its displayed features, every examination was rated on its own terms.

Fracture certainty was likewise classified according to maximum visibility in either projection to be able to aggregate information from both projections in trauma-oriented future analysis as well as to not exclude potential significant features unrecognizable to the human eye. Thus, in the frame of their examination, images were rated according to the full extent of the available patient data. Examinations were anonymized after image annotation.

### Image preparation

Images contained in the data set varied in size, resolution, field-of-view and brightness spectrum. In order to optimize evaluation by the network, an equalizing procedure for these image properties preceded consecutive randomized augmentation steps to provide homogenous training images.

**Fig. 2:**
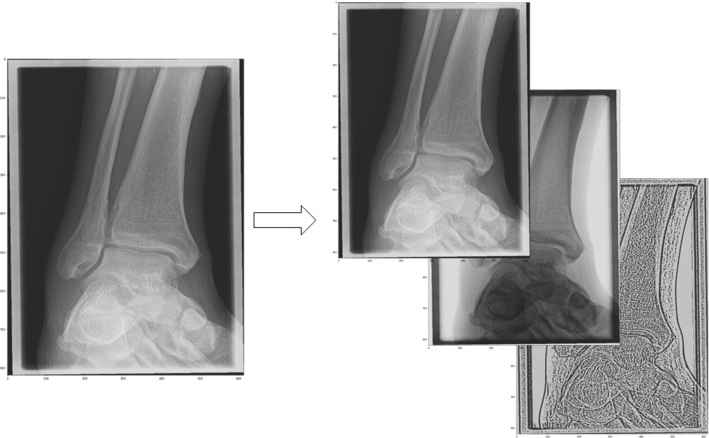
Image triplication for compatibility with the three-layered images in imagenet-pretrained network models. Inversion and edge-enhancing filters applied to second and third layers

**Fig. 3:**
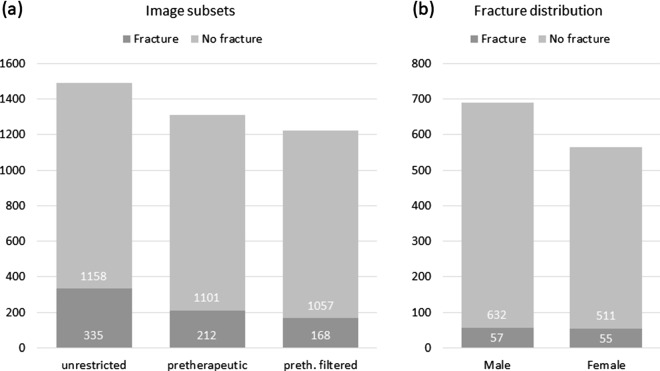
**a** Number of examinations and fracture distribution in image subsets **b** Examinations and gender specific fracture distribution

To this goal, the single-channel radiolucency values were normalized and cast to a spectrum of 8-bits. Images were resized and resampled to a uniform size of 1.6 times the input size of the neural network to minimize information loss during image augmentation. While medical x-ray images consist of one single channel of radiolucency values, digital encoding of color information commonly uses three channels in superposition. Such is the case for imagenet, a large database of classified two-dimensional natural images [10], where color layers are stored in three-dimensional arrays. To ensure compatibility with imagenet-pretrained networks, we embedded the x-ray images in three-dimensional arrays by filling the otherwise unused or redundant channels with filtered copies of the original image run through a brightness inversion filter in the second and an edge-enhancing filter (based on Adaptive mean thresholding) in the third channel (Fig. 2). For the training purposes presented here, eligible ap-views were randomly grouped into proportionally fixed-sized training (80%) and validation (20%) sets after completion of image annotation. The ratio was preserved for the image subsets used for specific training as described in the following, validation sets were not used for model training. In order to minimize effects of overfitting, we also ensured that all examinations of individual patients were assigned exclusively to the training or validation sets (Fig. 3).

### Comparative training, network and training protocol

We hypothesized improved prediction for models trained on images selected according to stricter feature criteria for their reduced amount of confounding features, and intended to quantify this by comparing selectively trained model performances using their area under the receiver operating characteristic curves (ROC AUC). Selective training was performed by defining training subsets according to the assigned labels of their images.

**Fig. 4:**
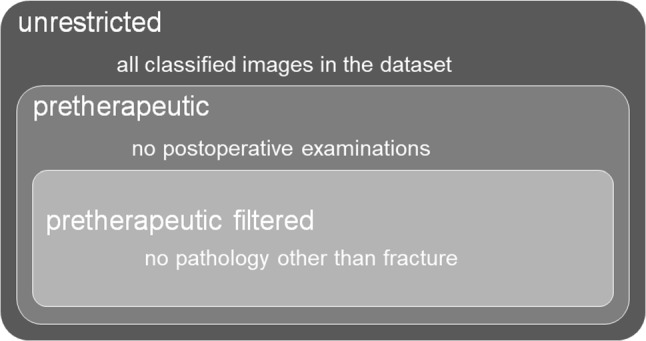
Training subset definitions

In particular, we intended to investigate the effect of visible signs of therapy such as osteosynthetic materials and plaster casts as indirect indicators of fracture, such that models trained on an image set consisting of all ankles (unrestricted set) were compared to those trained on a set defined by excluding images displaying therapeutic appliances (pretherapeutic set). Further excluding images displaying non-traumatic osseous lesions such as tumors or excessive degeneration (filtered set) enabled training of a “baseline” model on only normal and fractured ankles without other pathologies (Fig. 4). The analysis was extended by comparing model performances after successive inclusion of lower fracture certainty classes (fracture classes 0 vs 1, 0 vs 1–2, and 0 vs 1–3).

### Feature filtering was performed on both training and validation subsets for every model. Excluded examinations were not redistributed to other classes

An imagenet-pretrained Inceptionv3-network architecture was modified by removing the top layers and adding one two-dimensional global average pooling, dropout and dense convolutional layer each onto the network as provided by the Keras API [11, 12]. The output layer was defined as a dense layer using SoftMax-activation.

Network architecture, training runs and dataset augmentation were implemented using the Tensorflow 2.6 library [13]. Performances were monitored and recorded via Tensorboard by registering loss, categorical accuracy, precision, recall and ROC AUC for the training set. The initial learning rate was set to 0.1 and gradually decayed down to 0.005 over the course of training using a polygonal decay. We used Adagrad as a solver, performing no specific hyperparameter tuning.

Training runs were uniformly set to 200 epochs of 100 steps using a batch size of 15 images. In order to achieve equally-mixed training batches, label-based balancing was performed on batch generation, as well as data set augmentation. Augmentation consisted of random vertical image flipping, rotation of up to 20°, contrast adjustment and image cropping. Afterward, images were resized to an input size of 340 × 512 pixels, which was determined by taking into account the oblong shape of ankle radiographs as well as GPU memory capacity. All hyperparameters were set empirically and kept constant for all training runs. Validation results were calculated using integrated Tensorflow prediction functions and Scikit-learn 0.24.2. For training, we used NVidia GTX 1080 TI-GPUs on dedicated server machines.

## Results

As our total training dataset was biased in favor of its fracture prevalence, no epidemiologically valid conclusions can be drawn from it as is. For the unbiased subselection from 2017–2019, 1,255 patients were included. The male predominance of 689 vs 566 female patients suggests a predisposition of male gender to relevant trauma exposure and subsequent imaging diagnostics in the setting of radiographic imaging numbers. While in agreement with [14, 15] the proportion of fractures to examinations was higher in female patients, there was no significant gender bias (Fig. 3). Examination numbers by fracture certainty class are listed in Table 1.

Filtering images by non-fracture features as explained above produced the following subsets:

1. Unrestricted set: The initial image inclusion criteria yielded 1,493 images (335 fracture-positive, 1,158 fracture-negative).
2. Pretherapeutic set: Reduction to pretherapeutic x-rays yielded 1,313 images (212 fracture-positive, 1,101 fracture-negative).
3. Filtered set: Limiting the pretherapeutic images to those devoid of other bony pathology and foreign bodies lead to 1,225 studies (168 fracture-positive, 1,057 fracture-negative).

The results were compared between the described fracture certainty classes 1 to 3 and the fracture-negative class 0 cases (Table 1). On the unrestricted dataset, the imagenet-pretrained Inceptionv3-network achieved a maximum AUC of 0.904 for overall fracture detection on the validation set (class 0 vs classes 1, 2 and 3) with an accuracy (ACC) of 0.874. Narrowing the set to fractures of better discernibility by excluding doubtful (0 vs 1, 2) and faint fractures (0 vs 1) resulted in an AUC of 0.938 (ACC 0.884) and 0.943 (ACC 0.903), respectively. For all detection tasks, training on the pretherapeutic subset resulted in a higher AUC compared to training on the unrestricted subset. This relation proved robust to slight hyperparameter variations, whereas there was no consistent trend when comparing the models trained on the unrestricted to those trained on the filtered set. The complete results comparing the different datasets and subsets concerning fracture certainty are presented in Table 2.

**Table 2:** Model performances per subset and task

Validation set AUC (ACC)	Fractures 0vs1	Fractures 0vs12	Fractures 0vs123
Unrestricted	0.943 [0.903—0.964] (0.903)	0.938 [0.907—0.965] (0.884)	0.904 [0.872—0.934] (0.874)
Pretherapeutic	0.955 [0.926—0.979] (0.919)	0.954 [0.926—0.978] (0.895)	0.917 [0.870—0.948] (0.871)
Filtered	0.939 [0.909—0.968] (0.895)	0.946 [0.916—0.973] (0.883)	0.896 [0.850—0.937] (0.879)

Network performance in ROC AUC (ACC) for classification of fracture against no fracture: detection of certain fractures (0vs1), certain or doubtful fractures (0vs12) and certain, doubtful or possibly avulsed fractures (0vs123). 95% confidence intervals in brackets as calculated via bootstrapping (10,000 repeats) are not an indication for statistically significant differences between models, since training was performed on distinct subsets

## Discussion

In this study, we introduced a dataset of annotated ankle x-rays and demonstrated the effect of its multiclass labeling by performing network training on the task of automated fracture detection on distinct subsets of images, thus quantifying the influence of selected image features. By utilizing customized state-of-the-art preprocessing and augmentation methods on both the images themselves as well as the composition of the training data set, our study performed better compared to most contemporary ankle studies, including both convolutional neural networks [16, 17] and traditional machine learning methods [18]. For convolutional neural networks, the prevalent training protocol incorporates random dataset augmentation as performed in our study as well as triplication of the medical image for use on imagenet-pretrained networks.

Kim and colleagues achieved a mean AUC of 0.89 on ap-views using the pretrained Inceptionv3-network architecture without layer customization on a similarly sized dataset of 1226 examinations and a comparable augmentation scheme [16]. The ratio of positive to negative images was almost inverted with mostly fractured ankles (85% against 15%), which for a binary classification task should result in a comparable sampling bias. We attribute the increase in our AUC to the custom layers of the network as well as the additional information provided by the filtered images in the third dimension. Kitamura and colleagues reached an accuracy of up to 81% when combining both different ankle projections as well as different networks [17]. Utilizing the Inceptionv3-network by itself, the accuracy reached 74% for all three standard projections combined, 70% for a single view. Half of their dataset of 596 images were of fractures, eschewing the need for fine balancing during training. In contrast to our study, training was de novo, i.e., without pretrained models.

We noted no obvious correlation of high ROC AUC with high accuracy for our models, which we attribute mainly to the skewed balance of our initial image data set. Since accuracy is equal to the rate of true classifications, it would be suboptimal as a performance metric for fracture incidences at our institution, as models biased toward non-fracture would be favored. While accuracy is a valid measure of model performance, its higher dependence on pretest probability would explain the prevalence of the ROC AUC-metric in literature. However, in spite of our unbalanced set, our best performing models according to AUC also featured higher accuracies than those reported by Kitamura, indicating improved general performance.

In addition to the custom network layers and the triplicated filtered images, the training regimen itself differed in that it was de novo in contrast to our transfer learning approach. Although medical images differ from the natural images used for imagenet-pretraining, larger and more diverse dataset sizes in combination with training on x-ray images for fine-tuning an already well-trained network apparently leads to convergence on a more robust parameter minimum within the reasonable timeframe of our training protocols.

The level of results attainable using neural networks is indicated in a multicenter, multiregion and multinetwork study of fracture detection by Jones and colleagues with a total of 715,434 images (median 40,658 per region) containing about 12% fractures, which achieved an AUC of 0.983 for the ankle region without artificial data augmentation [19].

Given the obvious difference in training set size between this large-scale study with the number of images one order of magnitude higher than ours, we attribute the largest part of the difference in performance to quantity and quality of the training set, and consider the choice and rigorous training of the network the second largest factor. Additional factors include augmentation, pretraining status of the network and the duration of training. Comparing the performance difference of our study to those of comparable set sizes and network structure especially in light of this very different study, the effects of improved preprocessing, optimized network layers as well as refined image data augmentation methods become evident.

Utilizing the remaining two color channels by copying especially the edge-enhanced filtered image might have provided additional benefit, although the exact extent of this needs to be addressed in dedicated investigations. In general, isolating and emphasizing imaging features for easier identification by a human investigator is thought to improve network performance at least in terms of convergence speed as well, but to our knowledge, no relevant evidence has been published at the time of writing.

In this regard, our aim to achieve a high precision using several improvements over common practice on average-sized image datasets could contribute to this question.

In order to gauge the influence of distinct image features on fracture classification performance, we performed training runs with and without images containing the features in question.

When training on the pretherapeutic image set without therapeutic appliances, models consistently performed better than on the unrestricted data set. The reason for this behavior might be the follow-ups with and without visible fracture features leading to lower correlation of fracture with therapeutic materials. Limiting the set to pretherapeutic images removed the palpable, confounding feature set of implants and bandages, effectively reducing the variability of possible image features, presumably simplifying the classification task.

On the other hand, reducing the image feature set even more by excluding other pathologies in the filtered set might have decreased the variability of training image features to a degree that overall prediction performance was reduced again to the initial level of the model trained on the unrestricted set.

Limiting training to ap-projections of the ankle while having rated fracture certainty as maximum visibility on both ap- and lateral projections invariably leads to discrepancy, which we chose not to address for the results presented here.

On the one hand, to some degree, misclassifications in a sufficiently large and diverse data set serve to improve network robustness, as in principle, fracture and non-fracture status exist on a spectrum of possible pathologies with overlapping imaging and clinical features, so that imaging findings conceptually need to be differentiated from objective diagnosis. A desirable network prediction thus would recreate that degree of overlap so that the variety of possible morphologies can rather be accounted for during interpretation of said prediction. To what level additional findings like secondary fracture signs could serve to aid that interpretation on a network level needs yet to be examined.

On the other hand, we would argue that rating an objective property apparently not visualized on the rated image conceptually extends the prediction process over those features recognizable to the human reader, whereas implementing a decision logic for separately trained, self-contained network predictions on different views equals a potential limitation to the level of optimal human performance. To make full use of a machine’s potential to permit complementary and comprehensive interpretation, it would therefore be necessary to expose it to a more comprehensive set of information than that contained in a single view.

## Conclusion

In conclusion, the results presented here demonstrate the multidimensional nature of influence on neural network performance and the advantage of training on a dataset containing ratings of additional features instead of only those the detection of which it is intended to train. Systematic analysis of various subsets offers insight into the relations of distinct image features and ultimately may help training classification networks by optimizing their training protocol. To this end, we defined a multiclass dataset of ankle x-ray images and compared the performance of a neural network trained on it to contemporary models, where additional beneficial factors for classification performance on medical images could be identified in preprocessing and customization of network top layers.

We plan to expand our multidimensional dataset in size and maximize its use in creating a robust supportive diagnostic tool for ankle x-rays as well as explore the relations of distinct image and clinical features to further our own understanding of pathology detection in conventional musculoskeletal imaging.

## Supplementary Information

Below is the link to the electronic supplementary material.

Supplementary file1 (DOCX 13 KB)

## Data Availability

The codebase and the trained models from the presented work is available on reasonable request. The dataset is available on request within the limits of the European GDPR and corresponding local adaptions.
