# Supplementary material for: Multiclass datasets expand neural network utility: an example on ankle radiographs
Source: Int J Comput Assist Radiol Surg. 2023 Feb 2;18(5):819–26. doi: 10.1007/s11548-023-02839-9 (PMC10113347; doi:10.1007/s11548-023-02839-9)
Supplement: Supplementary file 1 — Supplementary file1 (DOCX 13 KB) [file 11548_2023_2839_MOESM1_ESM.docx]

Supplementary table 1:

| **Labels** | **Image features** |
| --- | --- |
| Fracture | See tab. 2 |
| Other osseous pathology | Tumours, old fracture, excessive degeneration |
| Soft tissue lesion | Swelling, emphysema |
| Bandage | Including plaster casts |
| Foreign body | Therapeutic (screws, plates, fixateurs) or other (intravenous lines, splinters) |
| Postsurgical state | Imaging signs or according to documentation |
| Clear space | Abnormal tibiofibular clear space |
| Regular joint configuration | Considering symmetry, joint gap and articular plane |

Classification labels for the ankle X-Ray dataset. All labels except for fracture label are binary.

Supplementary table 2:

| **Validation set**  **Confusion matrix** | **Fractures 0vs1** | **Fractures 0vs12** | **Fractures 0vs123** |
| --- | --- | --- | --- |
| Unrestricted | \| 204 \| 10 \| \| --- \| --- \| \| 16 \| 38 \| | \| 198 \| 16 \| \| --- \| --- \| \| 16 \| 46 \| | \| 203 \| 11 \| \| --- \| --- \| \| 25 \| 46 \| |
| Pretherapeutic | \| 202 \| 3 \| \| --- \| --- \| \| 16 \| 15 \| | \| 196 \| 9 \| \| --- \| --- \| \| 16 \| 18 \| | \| 196 \| 9 \| \| --- \| --- \| \| 23 \| 20 \| |
| Filtered | \| 193 \| 6 \| \| --- \| --- \| \| 18 \| 12 \| | \| 188 \| 11 \| \| --- \| --- \| \| 16 \| 16 \| | \| 192 \| 7 \| \| --- \| --- \| \| 22 \| 19 \| |

Confusion matrices containing true negatives and false positives (top rows left to right), false negatives and true positives (bottom rows left to right) for each model trained on fracture certainty class prediction in each subset.
